# Co-occurrence of Wilson's disease and Duchenne muscular dystrophy in a Chinese patient: a case report

**DOI:** 10.3389/fped.2025.1529725

**Published:** 2025-04-01

**Authors:** Jiawei Wang, Dandan Sun, Yu Wang, Mingjuan Fang, Xun Wang

**Affiliations:** ^1^ Department of Neurology, The Affiliated Hospital of Institute of Neurology, Anhui University of Chinese Medicine, Hefei, China; ^2^ Department of Neurology, Yueyang Hospital of Integrated Traditional Chinese and Western Medicine, Shanghai University of Traditional Chinese Medicine, Shanghai, China

**Keywords:** Wilson's disease, Duchenne muscular dystrophy, rare liver disease, inflammatory response, copper

## Abstract

Wilson's disease (WD) and Duchenne muscular dystrophy (DMD) are rare genetic diseases, and their co-occurrence is even rarer. Here, we describe our experience diagnosing a 6-year-old male Chinese patient presenting with an atypical phenotype and two genetic causative factors who was ultimately diagnosed with coexisting WD and DMD. We used a comprehensive and systematic evaluation of the patient's history, physical examinations, laboratory tests, and genetic testing to make the diagnosis. The patient was treated for one year with therapy to inhibit copper absorption and an anti-inflammatory treatment, and their condition remained stable. This case suggests that the inflammatory response could be a common pathogenesis between these two diseases. It also demonstrates the clinical efficacy of anti-inflammatory therapy for WD with DMD. Furthermore, this case illustrates the importance of taking a detailed history and performing thorough physical examinations to diagnose coexisting hereditary diseases.

## 1 Introduction

Wilson's disease (WD) is a rare autosomal recessive genetic disease with a global incidence of approximately 1/45000–1/35,000 (now, when conducting large molecular genetic studies, the frequency is calculated as 1/7026.) (1). WD is caused by a mutation of the *ATP7B* gene on chromosome 13, resulting in the inactivation of the ATP7B transporter protein, obstructing copper excretion in bile. Excess copper accumulates in the liver, brain, kidney, muscles, and other tissues, which can cause common liver diseases, cirrhosis, neuropsychiatric disorders, and, rarely, muscle lesions (2).

Duchenne muscular dystrophy (DMD) is a rare X-linked recessive muscular disease occurring in 4.6/100,000 male newborns (3). The loss of dystrophin, encoded by the *DMD* gene, is the main pathogenesis of DMD, resulting in progressively aggravated skeletal muscle weakness and atrophy, intellectual development disorders, and cardiomyopathy (4).

WD and DMD are rare diseases involving multiple systems. Diagnosing each becomes more challenging if they are both present in the same patient owing to the complexity of the clinical phenotype and the relative inexperience of clinicians in diagnosing and managing these diseases. Therefore, this report describes our experience diagnosing a 6-year-old Chinese patient with WD and DMD.

## 2 Case description

**Figure 1:**
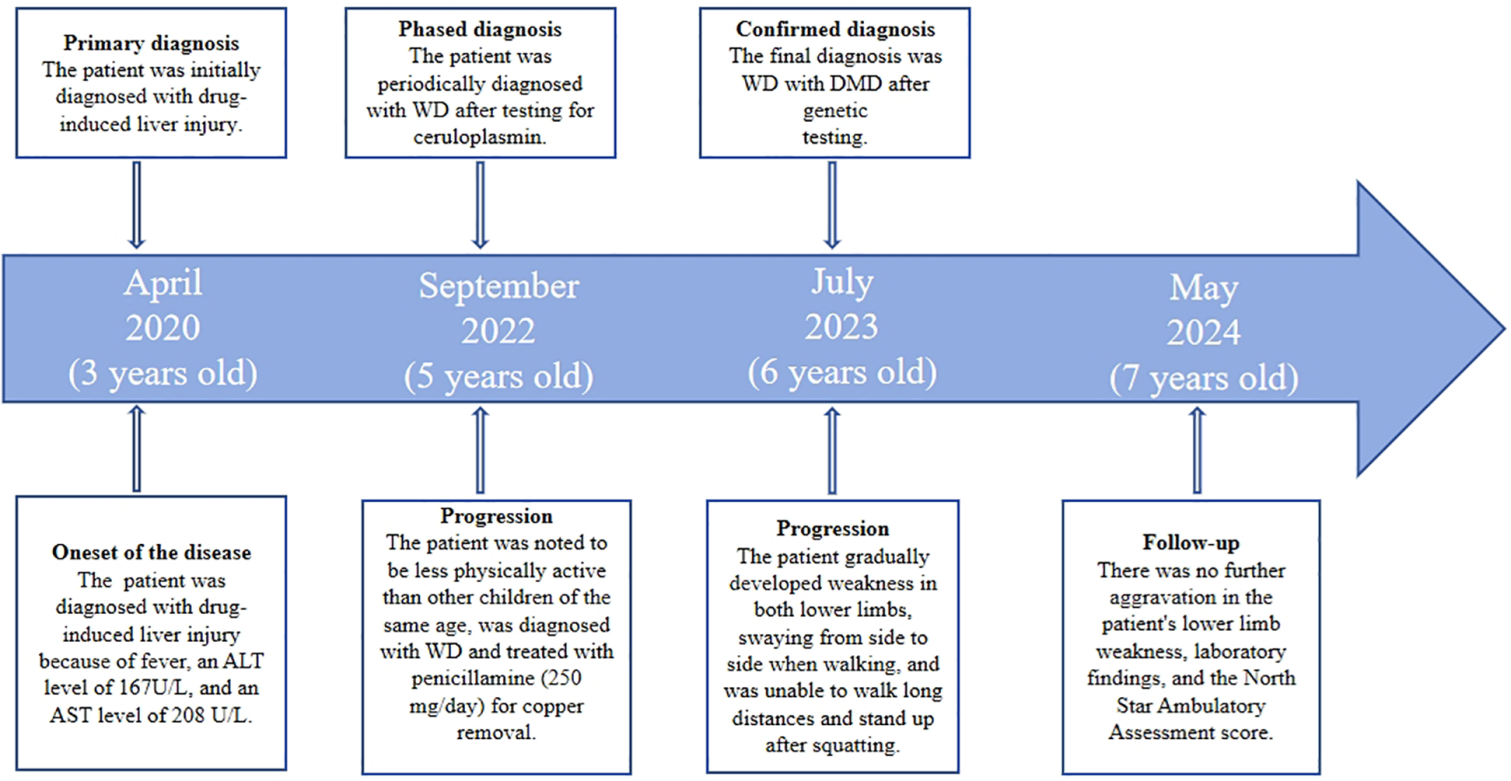
Case report timeline. ALT, alanine aminotransferase; AST, aspartate aminotransferase; DMD, duchenne muscular dystrophy; WD, Wilson's disease.

A 6-year-old male patient was admitted to our department in July 2023 due to elevated transaminase, weakness in both lower limbs, and difficulty squatting for three years. At 3 years old, the child was diagnosed with drug-induced liver injury at a local hospital because of fever, an alanine aminotransferase (ALT) level of 167 U/L, and an aspartate aminotransferase (AST) level of 208 U/L. At 5 years old, when the child was in kindergarten, they were noted to be less physically active (based on running and other physical activities) than other children of the same age. The patient was examined at a local hospital, and the liver function results were: ALT: 133 U/L, AST: 214 U/L, creatine kinase (CK): 12138 U/L, and ceruloplasmin (CP): 107 mg/L (reference, 200–600 mg/L). The patient was diagnosed with WD and treated with penicillamine (250 mg/day) for copper removal. At 6 years old, the patient gradually developed weakness in both lower limbs, swaying from side to side when walking, and was unable to walk long distances and stand up after squatting. The child's birth, past, personal, and family histories were unexceptional (see the timeline in Figure 1).

**Figure 2:**
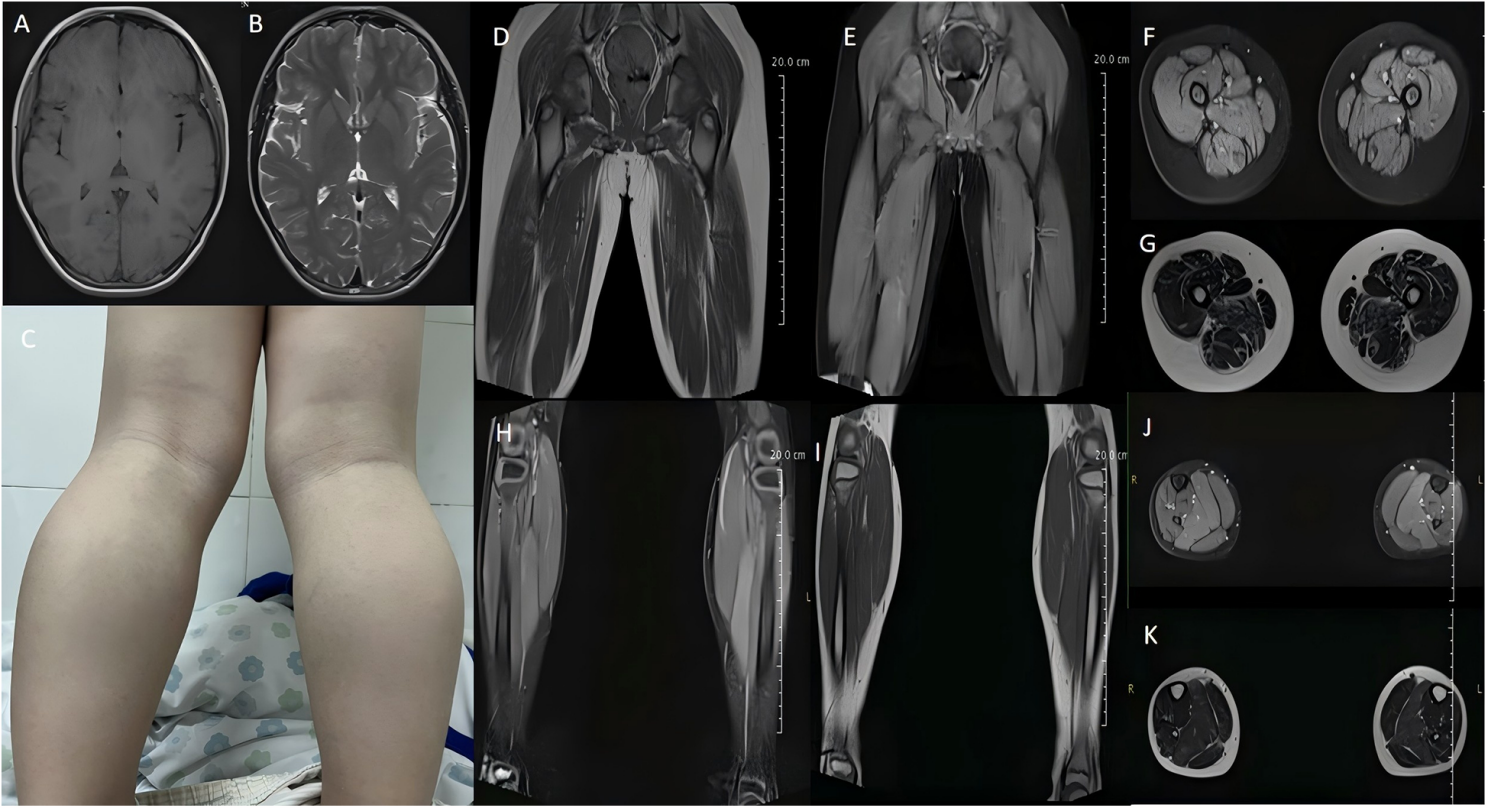
Cranial magnetic resonance imaging (MRI) shows no significant abnormalities **(A,B)**. The patient had bilateral calf muscle hypertrophy **(C)**. MRI of the thigh shows atrophy of thigh muscle groups on both sides, with slightly sparse muscle fibers, slightly enlarged intermuscular gaps, and increased intermuscular fat **(D–G)**. MRI of the calf shows muscle swelling in some muscle groups of the posterior calf group bilaterally **(H–K)**.

A physical examination found no obvious abnormalities in the heart, lung, spinal column, or abdomen. A neurological examination showed that the child had mental clarity and clear speech but walked with a “duck step” gait. A cranial nerve examination also showed no obvious abnormalities. The muscle strength of both upper limbs was grade 5, and both lower limbs were grade 4 proximal and grade 4+ distal. The muscle tone of all four limbs was normal, but the radial membrane reflexes and the knee tendon reflexes were weakened bilaterally, and Babinski's sign was not elicited bilaterally. Mild bilateral calf muscle hypertrophy with tenderness (−) (Figure 2C) and Gowers' sign (+) was also present. The Mini-Mental State Examination test score was 28, and the North Star Ambulatory Assessment score (NSAA) was 22 points.

Laboratory examinations showed the following: AST: 62 U/L, ALT: 80 U/L, CK: 6,985 U/L, CP: 148.0 mg/L, interleukin (IL)-6: 7.8 pg/ml, and tumor necrosis factor-alpha (TNF-α): 9.55 pg/ml (Table 1). The hepatitis virology results were negative. The basal urine copper level was 92.07 ug/24 h.

**Table 1:** Laboratory examination results of the 6-year-old male patient admitted for suspected Wilson's disease and Duchenne muscular dystrophy.

Age (years)	AST (U/L)	ALT (U/L)	CK (U/L)	CP (mg/L)	IL-6 (pg/ml)	TNF-α (pg/ml)	NSAA score (points)
3	208	167	–	–	–	–	–
5	214	133	12,138	107	–	–	–
6	62	80	6,985	148	7.8	9.55	22
7	51	50	2,793	155	4.2	4.94	25

AST, aspartate aminotransferase (0–50 U/L); ALT, alanine aminotransferase (0–50 U/L); CK, creatine kinase (24–170 U/L); CP, ceruloplasmin (200–600 mg/L); IL-6, interleukin-6 (<5.4 pg/ml); TNF-α, tumor necrosis factor-alpha (<16.5 pg/ml); NSAA, north star ambulatory assessment (Total scores: 33points).

Electrocardiographic and cardiac ultrasonographic findings were normal. Abdominal ultrasonography showed a slightly dense echogenicity in the hepatic region, and elastography showed no or mild liver fibrosis. Auditory and visually evoked potentials showed no abnormalities. Electromyography showed myogenic damage in the right biceps brachii, right deltoid, bilateral anterior tibialis, and bilateral internal femoral muscles. The patient was negative for the corneal Kayser-Fleischer ring (−). Cranial magnetic resonance imaging revealed no significant abnormalities (Figures 2A,B), but magnetic resonance imaging of top the thigh shows atrophy of thigh muscle groups on both sides, with slightly sparse muscle fibers, slightly enlarged intermuscular gaps, and increased intermuscular fat (Figures 2D–G); and magnetic resonance imaging of the calf shows muscle swelling in some muscle groups of the posterior calf group bilaterally (Figures 2H–K).

**Figure 3:**
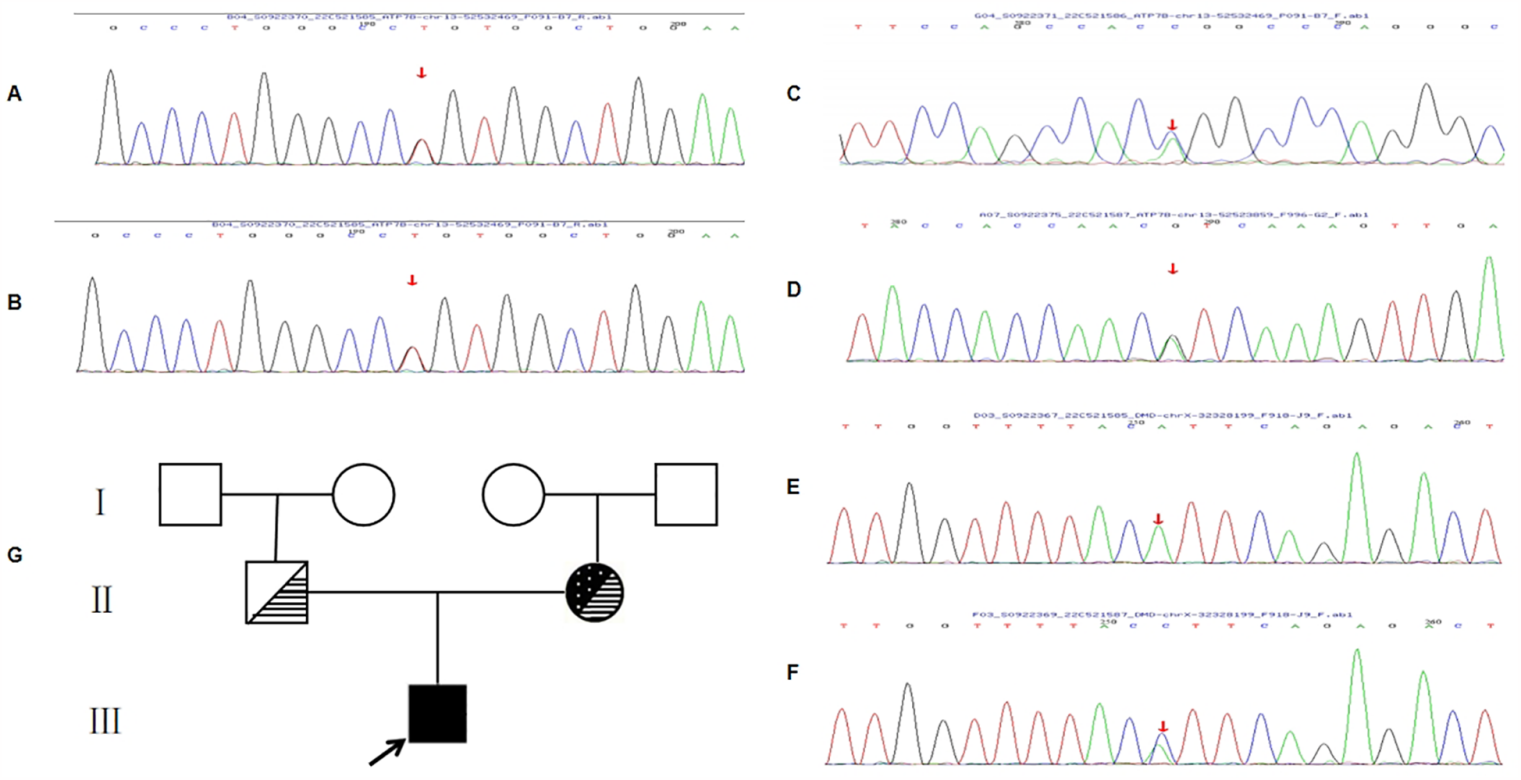
A mutational analysis of 21 exons and the flanking sequences of the *ATP7B* gene was performed via targeted sequencing and verified by Sanger sequencing. Compound heterozygous missense variants in *ATP7B* of pre-documented individuals at c.2333G > T (p.Arg778Leu) and c.2804C > T (p.Thr935Met) **(A,B)** are present, all of which are known pathogenic mutations. Lineage validation reveals that the variants originated from the father (c.2333G > T) **(C)** and mother (c.2804C > T) **(D)**. A mutation analysis of the exon and flanking sequences of the *DMD* gene was performed using targeted sequencing and validated by Sanger sequencing. The pre-documented patient had a c.6117G > T (p.Lys2039Asn) hemizygous missense variant in *DMD* with a known pathogenic mutation **(E)**. Lineage validation confirms that the variant originated from the mother (c.6117G > T) **(F)**. Pedigree of the family **(G)**. Lines within the circle or square indicate carriers of *ATP7B* gene, and white dots on a black background within the circle indicate *DMD* gene carrier. The black arrow indicates the proband.

The *ATP7B* gene had complex heterozygous variations of c.2333G > T (P.RG778Leu) and c.2804G > T (P.HR935Met) (Figures 3A,B). According to the American College of Medical Genetics (ACMG) guidelines, both mutation sites are pathogenic mutations, and a family verification analysis showed that both mutation sites originated from the patient's parents (Figures 3C,D). Additionally, c.6117G > T (p.Lys2039Asn) hemizygous missense variants were present in the *DMD* gene (Figure 3E), and the ACMG guidelines state that this mutation site is pathogenic. A phylogenetic analysis showed that the patient's mother had a heterozygous mutation at this site (Figure 3F).

## 3 Diagnostic assessment

The final diagnosis was WD (presymptomatic) with DMD. Thus, the patient was administered dimercaptopropanesulfonic acid (0.25 g IV) for 14 days during hospitalization to reduce the copper level. After discharge, the patient was treated with zinc gluconate (420 mg/d) to inhibit copper absorption, prednisone acetate (10 mg/kg/week) for anti-inflammation, and glutathione (0.3 g/d) for detoxification and anti-inflammation. The patient underwent a follow-up visit in May 2024 at 7 years old. Worsening of the bilateral lower extremity weakness was not observed, the NSAA score was 25 points, and the laboratory results were: AST: 51 U/L, ALT: 50 U/L, CK: 2,793 U/L, IL-6: 4.2 pg/ml, and TNF-α: 4.94 pg/ml.

## 4 Discussion

WD and DMD are rare genetic disorders, and their co-occurrence is even rarer, occurring in approximately 4.3% of patients diagnosed with a genetic disorder. In such cases, the child's phenotype might be atypical owing to the synergistic effect of the two genetic causative factors, which makes clinical diagnosis challenging. However, the prevalence of coexisting WD and DMD may be underestimated because neurologists tend to overlook elevated ALT levels caused by WD when treating patients with significantly elevated CK levels. On the other hand, DMD is often diagnosed based on elevated ALT and AST levels in liver function tests, which can be easily missed when there are no signs of muscle weakness. Therefore, it is easy to misdiagnose the disease as liver disease, prolonging the duration until a correct diagnosis.

Most individuals with WD develop symptoms between ages 5 and 35 years. Recognizing and diagnosing this treatable disease early is important because it can involve multiple systems and lead to diverse clinical manifestations. Most pediatric patients with WD present with abnormal liver function, making it imperative to evaluate any child with hepatic dysfunction since the early initiation of lifelong therapy is crucial to the patient's prognosis (5). The first symptoms of DMD appear between ages 2 and 3 years (6). The primary early manifestations are muscular atrophy and weakness of the proximal lower extremity and pelvic girdle, pseudohypertrophy of the calf gastrocnemius muscle, a “duck walk,” and Gowers' sign. In patients with no family history, DMD is usually diagnosed at ages 4–5 years (7). Generally, the condition is relatively stable before the age of 7 (6), after which the disease gradually progresses, resulting in difficulties in climbing stairs, falling, and generally losing the ability to walk autonomously around 12 years old.

In this case, the patient had markedly elevated ALT, AST, and CK levels but markedly reduced CP levels at an early stage. However, they were without symptoms; thus, they were diagnosed with WD. However, the cause of the elevated CK level was not further investigated until the patient developed signs and symptoms suggestive of progressive muscular dystrophy, such as bilateral lower extremity weakness and Gowers' sign positivity. In 2023, the patient was examined in our department, where we performed liver function, CP, urine copper, serum inflammatory marker, and electromyography tests, as well as gene analyses, where we found a p.(Arg778Leu) mutation on one allele and a p.(Thr935Met) mutation on the other of the *ATP7B* gene. Furthermore, we identified the patient had a deletion variant in exons 46–50 of the DMD gene, which confirmed the diagnosis of coexisting WD and DMD (8, 9).

WD and DMD can involve multiple systems; therefore, clarifying the relationship between these diseases is important. The inflammatory response is the body's most common pathological and physiological process, and WD and DMD have been associated with this response (10, 11). In this case, the patient had co-occurring WD and DMD, which resulted in abnormal copper metabolism, copper accumulation in the tissues, and cup-induced oxidative stress damage due to *ATP7B* gene defects, causing an inflammatory response (10). Levels of inflammatory factors in the serum, such as IL-6 and TNF-α, are abnormally high in patients with WD, attributed to the accumulation and abnormal metabolism of copper in the body, leading to oxidative stress and cellular damage, which triggers an inflammatory response and, consequently, further increases the serum levels of several inflammatory factors (12). A high copper level contributes to the inflammatory response that causes liver lesions (13); it can also trigger muscle lesions (14). Our patient had elevated serum inflammatory markers during their hospitalization in 2023 (IL-6: 7.8 pg/ml and TNF-α:9.55 pg/ml). Therefore, maintaining appropriate copper levels in the body is essential to suppress inflammation and reduce liver and muscle damage.

WD was one of the first liver diseases for which an effective drug therapy was developed (15), and its clinical prognosis is closely related to the timing of treatment. There is still no effective treatment for DMD, and the disease progresses rapidly, eventually causing disability and death. Therefore, finding an effective treatment for patients with DMD is critical. Glucocorticoids are the only treatment shown to slow the progression of DMD through their anti-inflammatory effects (16). Treatment is recommended to start at approximately 4 years old to minimize the rate of muscle function decline (17). For our patient, we administered zinc gluconate to inhibit copper absorption, prednisone (10 mg/kg/week) as an anti-inflammatory treatment (18), and preventative therapy against adverse hormonal reactions.

Motor symptom scores (e.g., the NSAA score) and the CK level in patients with DMD can directly and indirectly reflect the severity of the condition. In this case, after one year of treatment, the ALT, AST, CK, IL-6, and TNF-α levels decreased, the Mini-Mental State Examination score did not change, and the NSAA score decreased compared to the pre-treatment values, similar to a previous report (19). These changes were mainly characterized by an increase in muscle strength and a prolongation of the walking time; hormonal adverse effects did not occur. However, the patient is only seven years old, and the specific treatment plan must be adjusted based on the severity of the patient's clinical symptoms and the expected quality of survival, with multidisciplinary collaboration among the departments of neurology, hepatology, and rehabilitation to develop an optimal individualized plan and clinical efficacy follow-up assessment.

The co-occurrence of WD and DMD is exceedingly rare, and children with WD and DMD may present with an atypical phenotype, given the presence of two genetic causative factors. Therefore, clinicians should perform comprehensive and systematic assessments of the patient's history and physical examinations. As early as possible, whole-exome sequencing combining a variety of molecular genetic tests would be helpful for early diagnosis and treatment to improve their clinical prognosis. Here, we report the case of a Chinese patient who presented with both WD and DMD, highlighting a possible link between these two diseases. However, further clinical and basic research is required to better understand the disease-related mechanisms and associations.

## Data Availability

The datasets presented in this study can be found in online repositories. The names of the repository/repositories and accession number(s) can be found in the article/Supplementary Material.
